# Vectisol Formulation Enhances Solubility of Resveratrol and Brings Its Benefits to Kidney Transplantation in a Preclinical Porcine Model

**DOI:** 10.3390/ijms20092268

**Published:** 2019-05-08

**Authors:** David Soussi, Jérôme Danion, Edouard Baulier, Frédéric Favreau, Ysé Sauvageon, Valentin Bossard, Xavier Matillon, Frédéric Turpin, El Mustapha Belgsir, Raphaël Thuillier, Thierry Hauet

**Affiliations:** 1 UMR Inserm U1082, Inserm Nouvelle Aquitaine, F-86021 Poitiers, France; soussi.david@gmail.com (D.S.); jerome.danion@me.com (J.D.); edouard.baulier@etu.univ-poitiers.fr (E.B.); Yse.SAUVAGEON-JAGAILLOUX@chu-poitiers.fr (Y.S.); valentin.bossard988@gmail.com (V.B.); xav.matillon@gmail.com (X.M.); rathuillier@gmail.com (R.T.); 2 Faculté de Médecine et de Pharmacie, Université de Poitiers, F-86021 Poitiers, France; 3 Service de Chirurgie viscérale et endocrinienne, CHU Poitiers, F-86021 Poitiers, France; 4 Service de Biochimie et Génétique Moléculaire, CHU Limoges, F-87042 Limoges, France; frederic.favreau@unilim.fr; 5 Service de Biochimie, CHU Poitiers, F-86021 Poitiers, France; 6 Faculté de Médecine, Université Claude Bernard Lyon 1, 69100 Villeurbanne, France; 7 Service d’urologie et de chirurgie de la transplantation, Hospices Civiles de Lyon, 69003 Lyon, France; 8 BioCydex, 1 rue Georges Bonnet, 86000 Poitiers, France; fturpin@biocydex.com (F.T.); mbelgsir@biocydex.com (E.M.B.); 9 Fédération Hospitalo-Universitaire SUPORT, CHU de Poitiers, F-86021 Poitiers, France; 10 IBiSA Plateforme ‘plate-forme MOdélisation Préclinique—Innovation Chirurgicale et Technologique (MOPICT), Genesis, INRA, CS 40 052, 17700 Surgères, France

**Keywords:** ischemia reperfusion injury, transplantation, organ preservation, oxidative stress, resveratrol

## Abstract

Current organ shortages have led centers to extend the acceptance criteria for organs, increasing the risk for adverse outcomes. Current preservation protocols have not been adapted so as to efficiently protect these organs. Herein, we target oxidative stress, the key mechanism of ischemia reperfusion injury. Vectisol^®^ is a novel antioxidant strategy based on the encapsulation of resveratrol into a cyclodextrin, increasing its bioavailability. We tested this compound as an additive to the most popular static preservation solutions and machine perfusion (LifePort) in a preclinical pig model of kidney autotransplantation. In regard to static preservation, supplementation improved glomerular filtration and proximal tubular function early recovery. Extended follow-up confirmed the higher level of protection, slowing chronic loss of function (creatininemia and proteinuria) and the onset of histological lesions. Regarding machine perfusion, the use of Vectisol^®^ decreased oxidative stress and apoptosis at the onset of reperfusion (30 min post declamping). Improved quality was confirmed with decreased early levels of circulating SOD (Superoxide Dismutase) and ASAT (asparagine amino transferase). Supplementation slowed the onset of chronic loss of function, as well as interstitial fibrosis and tubular atrophy. The simple addition of Vectisol^®^ to the preservation solution significantly improved the performance of organ preservation, with long-term effects on the outcome. This strategy is thus a key player for future multi-drug therapy aimed at ischemia reperfusion in transplantation.

## 1. Introduction

Organ transplantation is currently the best treatment for end-stage organ failure, offering patients a better quality of life and improving public health [1,2]. The kidney is the most transplanted organ, with an ever-rising number of procedures—for instance, the number of transplantations in France reached 3615 in 2016 [3]. However, high as this may seem, this number is far below the actual need, with waiting lists now reaching a fivefold difference: again in France in 2016, the total number of registered patients reached 17,698. This shortage is worsening even though the acceptance criteria has been extended, leading to a majority of transplanted organs originating from “marginal” donors, i.e., high-risk donors such as DCD (Deceased after Circulatory Death) donors and extended-criteria cadaveric donors (ECD).

This profound alteration of donor demographics has important consequences in terms of outcomes. Indeed, organs from these donors are more sensitive to ischemia reperfusion (IR), and thus more prone to delayed graft function (DGF) and primary graft non-function (PNF) [4,5]. Optimization of storage conditions, such as the generalized use of Machine Perfusion (MP), permits a measure of reduction in the rate of these adverse events, but they unfortunately do remain high [6,7,8,9]. Hence, this method of organ preservation still requires improvement.

One of the hallmarks of current organ preservation methods is anoxia. Oxygen is primordial for several mechanisms within the cells, from the formation of disulfide bonds in the protein quaternary structure folding to electron acceptors in the red-ox reaction—however, its most critical form of usage takes place at the mitochondria respiratory chain level, where oxygen is the final acceptor of electrons that is released along the chain and driven towards complex IV, the only enzyme able to tetrareduce dioxygen into water. However, in the absence of this acceptor, the chain does not go silent [10], and uses the reduction of fumarate to generate reduced species to feed the chain; however, this process leads to the accumulation of succinate.

However, the difficulty arises when oxygen is re-introduced in the system at the reperfusion stage. Indeed, its captation of a single electron produces superoxide anion, the first reactive oxygen species (ROS) and source of oxidative stress. The main source of ROS is the succinate generated during ischemia, as above a certain threshold, the amount of electrons generated by complex II cannot be handled by complexes III and IV. Thus, a phenomenon called reverse electron transfer takes place, through which the excess electrons are transferred to complex I. Unfortunately, complex I cannot tetrareduce oxygen and only monoreduces it, producing superoxide anion. Oxidative stress and ROS production are thus one of the very first events that take place during reperfusion, with an intensity proportional to anoxia time. ROS targets the cell structure indiscriminately, from the DNA to proteins to lipids, leading to loss of function and permeability issues, which, above a certain threshold, induce cell death and the release of damage-associated patterns. ROS production is also mainly responsible for the destruction of the glycocalyx at the cell surface and, in consequence, lesions, among which are coagulation and sterile inflammation [11]. Oxidative stress is thus one of the main original culprits of IR lesion, being the source of a range of damaged mechanisms culminating in sterile inflammation.

Targeting oxidative stress at its source it thus key to properly preserving organs. Numerous strategies have been preclinically and clinically tested—at the donor, organ, and recipient levels—through the use of various techniques, such as preconditioning, machine perfusion, gases, and antioxidative molecules [12]. The main lessons from these studies appear to be a need for the bioavailability of compounds, as antioxidants are generally hydrophobic and poorly distributed with the need for versatility, guaranteeing the use in any preservation modality (e.g., static versus machine perfusion), and long shelf-life. Strong preclinical demonstration of efficiency is also a critical factor before going from the bench to the bedside.

Herein, we demonstrate the benefits of using Vectisol^®^, a resveratrol-cyclodextrin conjugate which permits increased bioavailability of the well-known antioxidant molecule [13] in a highly stable formulation in kidney transplantation.

**Figure 1:**
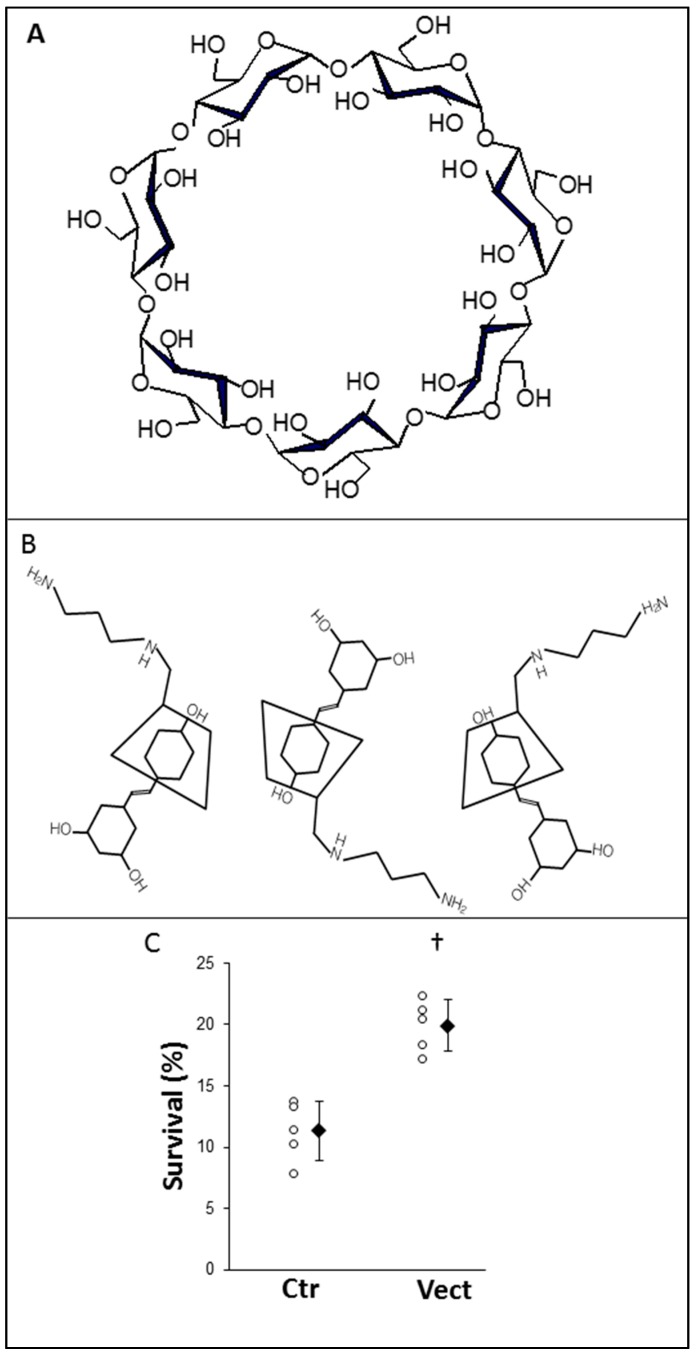
Vectisol^®^ chemical composition, rationale, and in vitro effectiveness. (**A**) Molecular structure of β-cyclodextrin with seven glucose subunits. (**B**) Schematic representation of the Vectisol^®^ inclusion complex. (**C**) Cells were subjected to conditions mimicking organ preservation, using a 24 h hypothermia/hypoxia incubation with University of Wisconsin solution, during which Vectisol^®^ was added in the Vectisol group, followed by a return to normal culture conditions, at which point the cell survival was assessed. Shown are each data point, as well as mean ±SD, statistics (Wilcoxon Test): †: *p* < 0.05 to Ctr.

The low solubility of trans-resveratrol and its propensity to organize in molecular “stacks” greatly reduces its bioaccessibility. In Vectisol^®^, the solubilization of trans-resveratrol is provided by a cyclodextrin, able to form complexes by taking the hydrophobic part of a molecule into their cavity (Figure 1A). Formation of the so-called “inclusion complex” increases the aqueous solubility and/or the stability of the molecule and enhances its bioavailability. The inclusion complex is formed without any covalent bond creation, and a dynamic equilibrium takes place between the CD and the hydrophobic molecule, M:

M+CD⇌[M:CD]

Hydrophobic molecules are thus released from the complex according to a constant of complexation.

The β-cyclodextrin (BCD) cavity size is perfectly designed to form an inclusion complex with trans-resveratrol, but has very limited solubility. Otherwise, it has been shown that substitution of at least one hydrogen of the primary hydroxyl groups of the BCD results in drastic improvement in its aqueous solubility. In this study, we developed a trans-resveratrol carrier, namely, a β-cyclodextrin derivative with improved inclusion capacity and solubility. This is a monomodified β-cyclodextrin which is nontoxic, nonhemolytic, and easily soluble at the physiological pH and, in addition, capable of exerting oncotic pressure according to mechanisms combining the properties of impermeant, such as mannitol or raffinose, and colloids (e.g., Hydroxyethyl Starch (HES), Polyethylene glycol (PEG), dextran). Monopropanediamino-β-cyclodextrin (MPDA-BCD), thanks to its positive charge, may also interact with the negative charge at the membrane cell.

Our investigation spans the most frequently used organ preservation modalities, from static preservation with multiple solutions to machine perfusion.

## 2. Results

### 2.1. In Vitro Assay of Vectisol^®^ Benefits

Primary kidney cortex endothelial cells were subjected to conditions mimicking organ preservation and reperfusion (Figure 1). Cell survival in regular conditions induced an important level of cell death (approximately 90%), while simple supplementation with Vectisol^®^ was able to double the survival rate in a significant manner (*p* = 0.009).

### 2.2. Static Preservation Study

#### 2.2.1. Early Function Recovery

**Figure 2:**
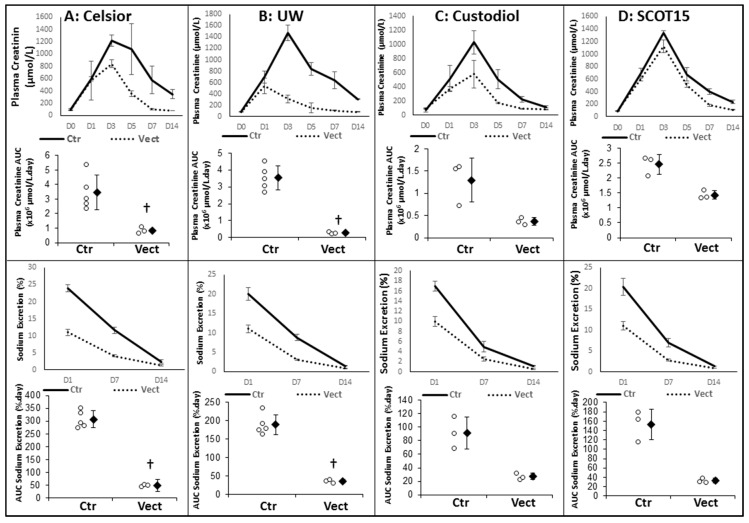
Evaluation of Vectisol^®^ benefits during static preservation: early follow-up. Vectisol was used to supplement preservation solutions in a porcine model of kidney autotransplantation, where solutions used were: (**A**) Celsior^®^; (**B**) University of Wisconsin^®^ (UW); (**C**) Custodiol^®^; and (**D**) Solution pour la Conservation des Organes en Transplantation (SCOT15^®^). The benefits were evaluated over the first two weeks post-reperfusion. Shown are: **Top**: plasma creatinine, **Bottom**: sodium excretion. Graphical evolution over the first two weeks is displayed, and AUC (Area Under the Curve) comparison was used to determine the statistical difference. Shown are each data point (empty dots), as well as mean ± SD (filled diamonds), statistics (Wilcoxon Test): †: *p* < 0.05 to Ctr.

Function recovery immediately after reperfusion was evaluated through the measurement of serum creatinine levels (assaying glomerular filtration) and sodium excretion (assaying tubular function). We determined that the different solutions used displayed similar serum creatinine patterns: rising after reperfusion and peaking at Day 3 between 1000 and 1400 µmol/L, followed by a recovery of function, sometimes partial, until Day 14. Sodium excretion also showed similar evolutions, starting from approximately 20–25% at Day 1 and reaching normal levels (<5%) by Day 14. Supplementation of the preservation solution was beneficial overall, though with some dissimilarities—with Celsior^®^ (Figure 2A), the pattern was similar but the creatininemia peak was lower (≈800 µmol/L), and recovery was faster, where the sodium excretion started lower (≈10%) as well. AUC (Area Under the Curve) comparison for both parameters showed statistical significance (*p* = 0.036 for both). With University of Wisconsin^®^ (UW) (Figure 2B), the effect was more intense, with a shorter time to peak in serum creatinine (Day 1 vs. Day 3), where AUC analysis again showed statistical differences (*p* = 0.036 for both). The benefits of Vectisol^®^ supplementation were also evident in the Custodiol^®^ (Figure 2C) and Solution pour la Conservation des Organes en Transplantation^®^ (SCOT15) (Figure 2D) groups, particularly in the formed version, with clear trends toward smaller AUC for both creatininemia and sodium excretion. The animal number was too low for statistical analysis.

The effect of the treatment was also measured at the tissue level, through histological evaluation of loss of brush border and endoluminal tubular cell detachment at Day 7 (Table 1). Results show that these injuries were important in the control animals, and while Vectisol^®^ supplementation did not benefit the kidneys in terms of brush border loss, the recorded level of endoluminal detachment was consistently lower in the treated groups, reaching significance in the Celsior^®^ and UW groups (*p* = 0.030 and 0.036, respectively).

#### 2.2.2. Chronic Outcome (Figure 3)

The chronic outcome was first evaluated at the end of the follow-up (1 month) through the glomerular function, using serum creatinine and proteinuria measurements. Overall, creatininemia was high in the untreated groups, reaching 250–300 µmol/L in the Celsior^®^ and UW groups; and proteinuria was also important, particularly in these same groups (reaching two or more g/24 h). Supplementation of the solution was consistently permitted to kidney function in a more effective way, with serum creatinine values reaching normal levels in the pig (80–100 µmol/L) and proteinuria remaining below 0.2 g/24 h. Both the Celsior^®^ and UW groups showed statistically significant effects of the treatment (*p* = 0.037). The animal number was too low in the Custodiol^®^ and SCOT^®^ groups for statistical analysis.

To finalize the analysis, histopathological analysis with HES and Sirius Red staining was performed on samples from the UW group, determining that control animals had high levels of tubular atrophy (1.3 ± 0.4%) and interstitial fibrosis (15.0 ± 5.5%), whereas kidneys preserved with solution supplemented by Vectisol^®^ showed lower values for both parameters (tubular atrophy = 0.2 ± 0.1; interstitial fibrosis = 2.0 ± 1.5%).

### 2.3. Machine Preservation Study

#### 2.3.1. Post-Reperfusion Apoptosis and Oxidative Stress

To test the ability of Vectisol^®^ to protect the graft during machine preservation, we used a preclinical model of kidney transplantation in the pig, in which kidneys were preserved in the LifePort^®^ machine using the recommended solution (KPS), either supplemented or not with Vectisol^®^.

**Figure 4:**
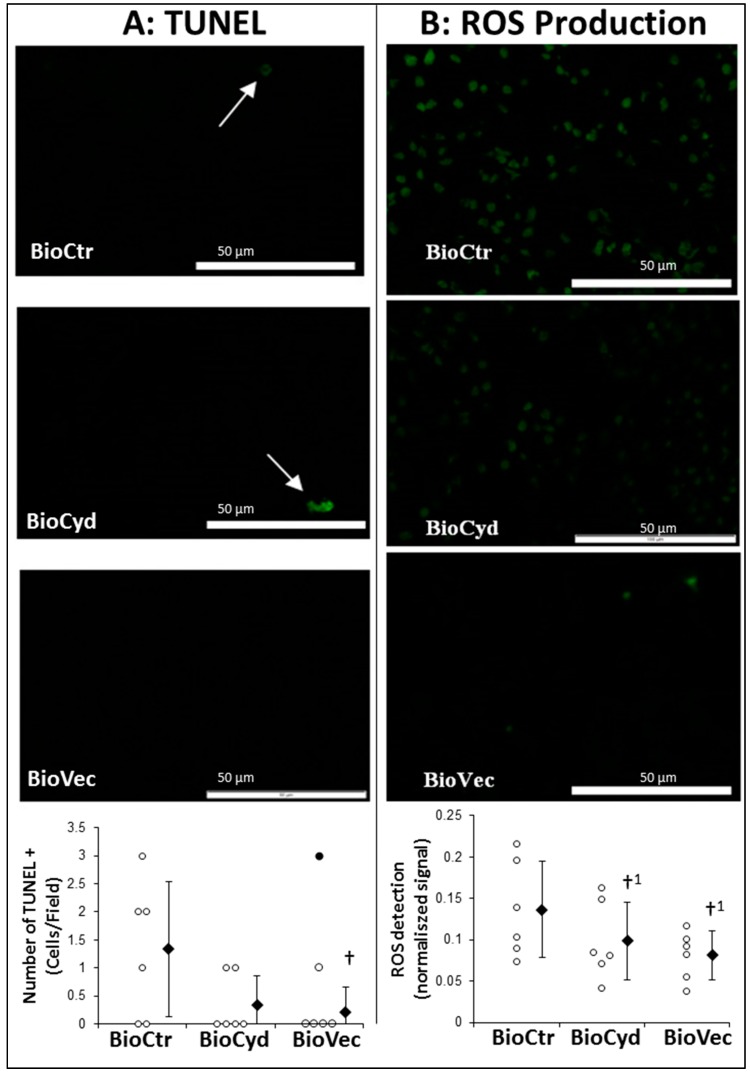
Evaluation of Vectisol^®^ benefits during machine preservation 30 min after reperfusion. Cortical biopsies were collected 30 min after reperfusion and snap-frozen in an OCT (Optimal Cutting Temperature) compound. Samples were processed for TUNEL (**A**) or CellRox (**B**) staining, and signals were quantified as exposed in the methods. Representative images for each group are shown, and quantification of signals for all animals is presented at the bottom. Shown are each data point (empty dots) as well as mean ± SD (filled diamonds), statistics (Dunn’s Test followed by Kruskal Wallis post-hoc): †: *p* < 0.05 to BioCtR, †^1^: *p* < 0.05 to BioCtr one-sided test; black dots are outliers.

We first measured the impact of the treatment on apoptosis and oxidative stress, using biopsies collected 30 min after reperfusion. TUNEL (terminal deoxynucleotidyl transferase dUTP nick end labeling) labelling (Figure 4A) revealed that, compared to a detectable number of apoptotic cells in the control condition (BioCtr), treatment with either cyclodextrins alone (BioCyd) or Vectisol^®^ (BioVec) reduced this number, reaching significance in the BioVec animals (*p* = 0.032). Cell ROX staining (Figure 4B), which fluoresces when reduced by a ROS, indicated that post-reperfusion oxidative stress was important in the kidney, and that treatment with either formulations was able to reduce the amount of ROS detected, with lowest values recorded in the BioVec group.

#### 2.3.2. Evaluation of Early Lesion and Function

**Figure 5:**
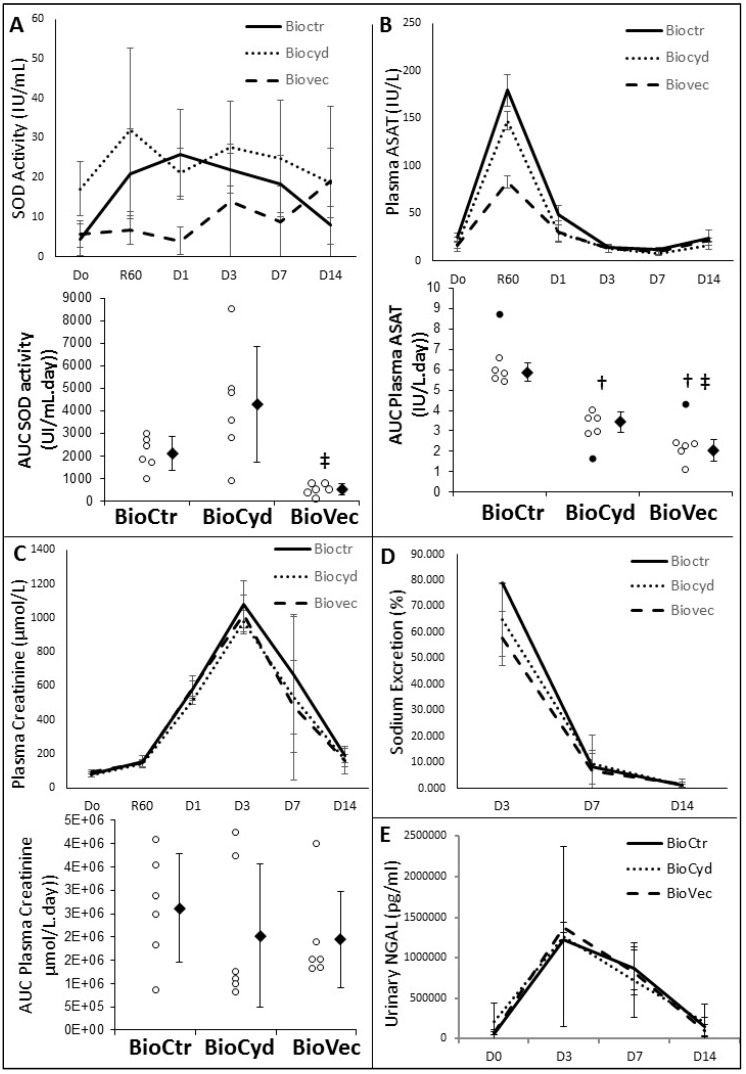
Evaluation of Vectisol^®^ benefits during machine preservation: early follow-up. The benefits of supplementation were evaluated during the first two weeks of transplantation. Several pathways were investigated: (**A**) Oxidative stress, through plasma superoxide dismutase (SOD); (**B**) cell loss, with asparagine amino transferase (ASAT); (**C**) glomerular filtration, using serum creatinine; (**D**) tubular function, through sodium excretion, and (**E**) kidney necrosis, through Neutrophil gelatinase-associated lipocalin (NGAL). Graphical evolution over the first two weeks is displayed, and AUC comparison was used to determine statistical differences when applicable. Shown are each data point (empty dots), as well as mean ± SD (filled diamonds), statistics (Dunn’s Test followed by Kruskal Wallis post-hoc): †: *p* < 0.05 to BioCtR, ‡: *p* < 0.05 to BioCyd; black dots are outliers.

To assess the importance of systemic oxidative stress, plasma levels of superoxide dismutase (SOD) were measured in the three groups (Figure 5A). While the standard deviation of the measure was important, likely due to the fact that we evaluated systemic, rather than organ-specific SOD activity, in both BioCtr and BioCyd groups, SOD activity increased during the first week, with a return toward Day 0 levels at Day 14. On the other hand, BioVect animals showed reduced SOD activity in the early days post-transplantation, with some increase recorded afterward. AUC analysis demonstrated that Vectisol^®^ supplementation showed lower overall SOD activation compared to the other groups (*p* = 0.12 vs. BioCtr and *p* = 0.0002 vs. BioCyd).

Tissue lesion in general was assessed through measurement of circulating asparagine amino transferase (ASAT) activity (Figure 5B). In the BioCtr animals, ASAT levels peaked (≈180 IU/L) as early as 60 min post-reperfusion and returned to normal levels by Day 3. Use of both cyclodextrins (*p* = 0.038) and Vectisol^®^ (*p* = 0.0002) both showed beneficial effects on ASAT detection, reducing the level of the peak, particularly in the BioVec animals, and being significantly more effective than cyclodextrin alone (*p* = 0.039).

Function recovery after reperfusion was evaluated through serum creatinine (glomerular filtration, Figure 5C) and sodium excretion (tubular function, Figure 5D). Moreover, kidney cellular necrosis was evaluated through measurement of urinary Neutrophil gelatinase-associated lipocalin (NGAL, Figure 5E). Overall, while the values recorded were lower for the BioVec group, differences were not significant.

#### 2.3.3. Evaluation of Chronic Function and Lesions

**Figure 6:**
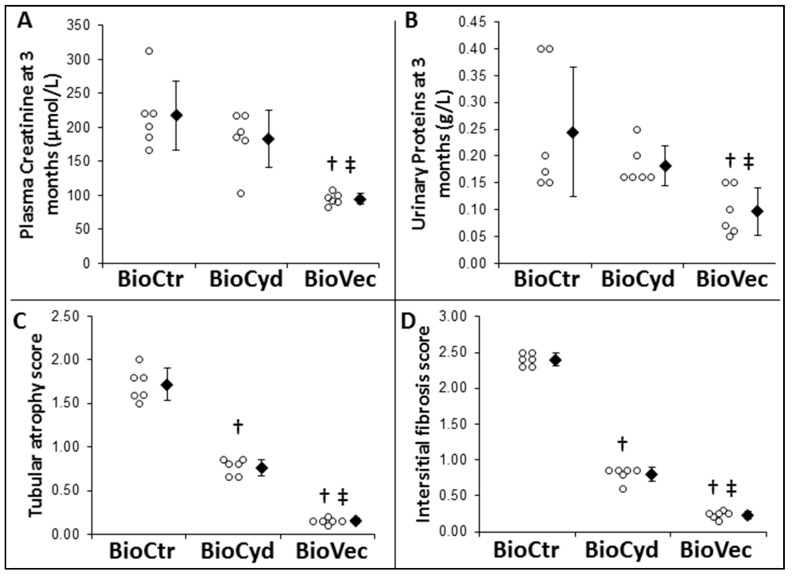
Evaluation of Vectisol^®^ benefits during static preservation: late follow-up. The benefits of supplementation were evaluated at the end of the three months’ follow-up. Biological evaluation was performed through (**A**) Plasma Creatinine and (**B**) Urinary Proteins. Histological evaluations of (**C**) Tubular Atrophy and (**D**) Intersitial Fibrosis was performed by a blinded pathologist following a semi-quantitative grading of the percentages of lesions by field: scores: 0 = no alteration, 1 = lesions < 25%, 2 = lesions between 25–50%, 3 = lesions between 51–75%, 4 = lesions between 76–100%. Shown are each data point (empty dots), as well as mean ± SD (filled diamonds), statistics (Dunn’s Test followed by Kruskal Wallis post-hoc): †: *p* < 0.05 to BioCtR, ‡: *p* < 0.05 to BioCyd.

To determine the long-term effects of the treatment, animals were followed for three months. Kidney function was evaluated through serum creatinine (Figure 6A) and proteinuria (Figure 6B). In the control group, plasma creatinine at the end of the follow-up was elevated (≈200 µmol/L) with increased proteinuria (≈0.2 g/L). Supplementation of the machine preservation solution with cyclodextrins alone was not beneficial; however, use of Vectisol^®^ provided a beneficial effect, translating into improved function at three months with reduced levels of creatininemia (≈100 µmol/L, *p* = 0.0005 vs BioCtr and *p* = 0.008 vs BioCyd) and proteinuria (≈0.1 g/L, *p* = 0.002 vs BioCtr and *p* = 0.003 vs. BioCyd).

**Figure 7:**
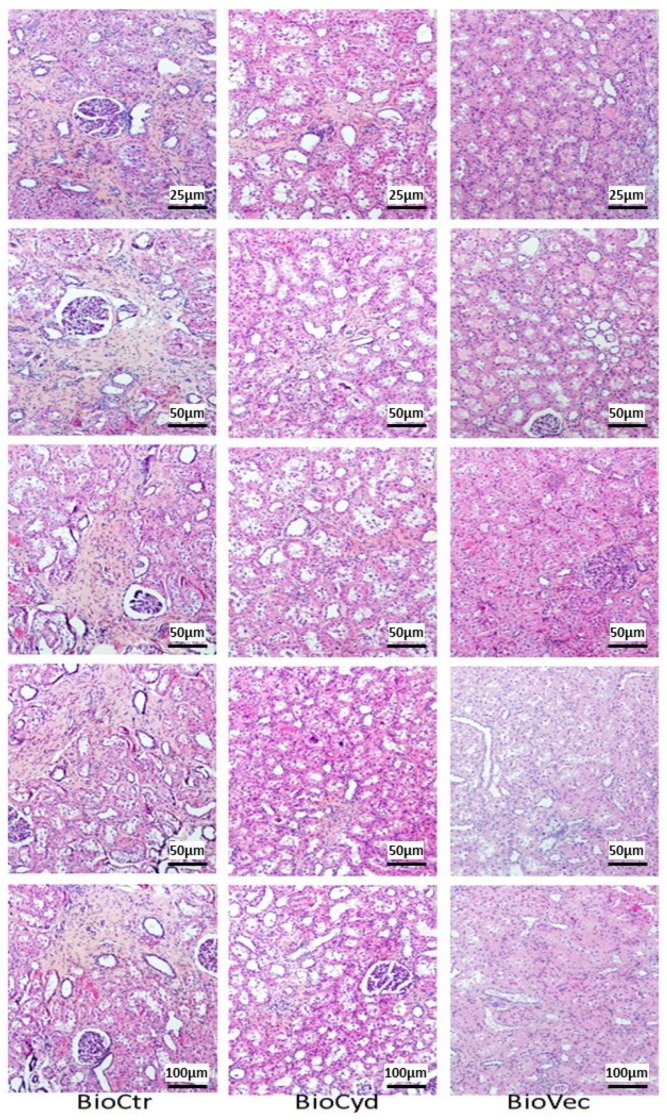
Representative images of histology at the end of the follow-up. Animals were euthanized after three months’ follow-up and cortical samples were formalin fixed and processed for PAS (Periodic Acid Schiff) staining. Each column represents a group, displaying several areas of the sample at the indicated magnifications.

To finalize the evaluation of chronic outcomes, we performed histological analysis on kidney cortical samples for tubular atrophy and interstitial fibrosis (Figure 7), two of the most common features of chronic graft loss. The score for both of these lesions was high in the BioCtr group (≈2–2.5), whereas kidneys preserved with cyclodextrins alone demonstrated significantly reduced scores (≈0.75 for tubular atrophy, *p* = 0.025, and ≈0.9 for interstitial fibrosis *p* = 0.025). However, the lowest levels were recorded in the kidneys of animals transplanted with solutions supplemented by Vectisol^®^ (≈0.1 for tubular atrophy, *p* < 0.000 to BioCtr and *p* = 0.025 to BioCyd, and ≈0.2 for interstitial fibrosis, *p* < 0.000 to BioCtr and *p* = 0.025 to BioCyd).

## 3. Discussion

In 2018, WHO (World Health Organization) estimated the number of kidney transplantations worldwide to be close to 80,000 (obtained from data of the ONT-WHO (Organización Nacional de Trasplantes-World Health Organization) Global Observatory on Donation and Transplantation), which only covered the needs of approximately 10–25% of the patients on waiting lists. The current trend of increased use of marginal organs is not temporary, but instead will only grow towards more at-risk strategies, and subsequently, the quality of organs to be transplanted will continue to decrease. This evolution requires a reformulation of organ preservation methods. Recently, it has been shown that extended cold ischemia time durations is directly related to the occurrence of adverse outcomes [14], which highlights the now-acknowledged importance of ischemia reperfusion in deciding the fate of the organ. It is thus of paramount importance to address the mechanisms underlying this lesion, among which oxidative stress is central.

Resveratrol is a polyphenol found in numerous plants, among which things such as the well-documented health benefits of grapes have been linked to the French Paradox [15]. Indeed, several studies have highlighted its anticancer activities, as well as effects on energy metabolism [16], vascular homeostasis, and NO-level regulation [17], and its most-documented activities against oxidative stress, either directly through free radical scavenging [18,19] or indirectly protecting against membrane oxidation [20]. From a mechanistic point of view, several pathways have been implicated in the response to Resveratrol, among which SIRT1 [21,22] has also been shown to have direct inhibitory effects on many kinases [23], as well as activating effects on others (such as AMP-activated protein kinase (AMPK)) [21,24] and inhibition of cyclooxygenases [25] and quinone reductase 2 [26]. It also has been implicated with signaling through the estrogen receptor [27] and the aryl hydrocarbon receptor [28].

We tested the benefits of Vectisol^®^ in a porcine model of kidney transplantation. The pig is an animal particularly well-suited for the study of transplantation, as it is anatomically very similar to humans, especially in regard to the multipapillary nature of its kidney and the vascular complexity stemming from it [29]. We first investigated supplementation of the preservation solution with a single dose of the compound during static preservation. This mode of preservation, although less protective than machine perfusion, remains the most-used transport modality worldwide, as MP is plagued by budgetary and logistical constraints. To further the relevance of our findings, we tested four of the most popular preservation solutions currently in use: University of Wisconsin^®^, the most distributed solution in kidney transplantation; Custodiol^®^, also known as Histidine Tryptophane Ketoglutarate (HTK), the second most-sold solution; Celsior^®^, used preferentially for heart protocols but sometimes found in that for kidneys; and SCOT15^®^, representing fourth-generation solutions, which are generally designed to address the ionic imbalance induced by ischemia and rely on polyethylene glycol as a colloid to decrease risks associated with historical colloids, such as hydroxyethyl starch, and profit from its immunocamouflage properties [30,31]. These were used in a standard model of autotransplanted porcine kidney, allowing us to assess the impact of ischemia reperfusion and the methods to alleviate it without the bias of immune suppression.

In this model, the effect of Vectisol was evidenced in the early phase post-transplantation, with significant improvements in terms of function, both regarding glomerular filtration and tubular function. While differences in the depth of the effect was observed between the solutions, an overall reduction in serum creatinine and sodium excretion AUC was observed, indicating that the stresses of IR were better managed at the tissue level, and cells were able to resume function faster and/or more effectively. The notable exception was SCOT15^®^, for which improvement of serum creatinine AUC was more modest; however, the effect on sodium excretion and the long-term benefits (highlighted below) validate the rationale of using Vectisol^®^ with this solution. In terms of histopathological evaluation of treatment benefits, we surprisingly found that treatment kidney displayed a high level of loss of brush border, while simultaneously showing less endoluminal detachment at Day 7. The bush border is a characteristic of the well-differentiated tubular cell, making it better able to perform its function. Increased loss at the seventh day, a time at which sodium reabsorption returns to normal in all groups, may perhaps indicate that rather than having lost the brush border, the cells in the Vectisol^®^ groups have yet to fully differentiate, as they are issued from a more active regenerative process. As cell loss was lower, such a hypothesis may be well-founded; however, more experiments focusing on the dynamics of repair between treated and untreated organs will need to be performed.

Long-term benefits were highlighted through the follow-up of our animals for 1 month. From a biological point of view, serum creatinine and proteinuria were all normal in the treated groups, the former being at the level found in healthy animals with two kidneys. Deterioration of function, highlighted in the untreated groups, is a precursor to chronic graft loss, and confirms the observations regarding the role of IR in setting the fate of the organ in the long-term. The ability of Vectisol^®^ to protect against this injury were further validated by histological analysis of chronic graft loss lesions, interstitial fibrosis and tubular atrophy, where both were significantly slowed in their advance in the treated kidneys.

We completed our demonstration on the benefits of Vectisol^®^ in another model of kidney transplantation, closer to the conditions found in marginal organ donation. This model was successfully used by our team to demonstrate the benefits of MP during kidney preservation [32], as well as to investigate the mechanisms underlying its benefits [33]. In this model, the kidney is subjected to 60 min warm ischemia prior to collection, a protocol which we determined increases the level of IR and profoundly affects transplantation outcomes [34,35]. As marginal donor organs are generally preserved by MP, we also adopted this strategy so as to validate the compatibility of Vectisol^®^ with machine perfusion. In the case of kidney MP, the choice of strategy is more limited, as the Lifeport device is also ubiquitously used. Thus, only two groups were used.

The benefits of Vectisol^®^ were highlighted as early as 30 min post reperfusion, with a measurement of oxidative stress at the tissue level. Resveratrol is mainly known for its antioxidative properties, and indeed, we observed a significant lowering of ROS production within the treated organs during reperfusion, the most damaging event in the early phase of transplantation. This reduction had a measurably positive impact on cell death, as evaluated by the TUNEL assay. A follow-up of the organs during the early phase of transplantation confirmed these findings, as several systemic markers did indeed indicate lower ROS production (SOD measurement) and cell death (ASAT). While SOD level showed important deviation, likely due to the fact that we evaluated systemic, rather than organ-specific SOD activity, inter-sample variation was hence more important, as ACU revealed the differences between the groups which reached significance. This protection against oxidative stress and its consequence on cellular health appeared to translate into better function recovery, as patterns of plasma creatinine and sodium excretion suggested improvements in the Vectisol^®^ group. However, as is often the case when using two protecting strategies (herein, Vectisol^®^ and MP), the effects were sometimes not additive, and in our observations, we could not significantly highlight the benefits of the treatment in the early post-transplant phase. Further evaluation of necrosis with urinary NGAL (Neutrophil Gelatinase-Associated Lipocalin), a more sensitive marker of kidney injury [36], did not permit us to discriminate further, confirming the non-additivity of the protection provided with each strategy. This suggests that either more sensitive markers must be used, such as KIM1 [37] or Vanin-1 [38,39], or a more discriminant model [29]. Further investigations will be required to explore these possibilities.

We thus focused on chronic outcomes whilst following the animals for three months. Being three times longer than the static follow-up, it permitted us to increase the difference between the untreated and treated groups suggested by the early outcome markers. Indeed, the biological markers of kidney function degradation, serum creatinine and proteinuria, were both increased in the MP-alone groups, whereas levels for these markers were significantly lower in the Vectisol^®^ group, indicating that the kidney retained the integrity of the glomerular filter despite the important level of injury which was withstood during transplantation. This was confirmed by histological analysis, revealing that even with MP, the kidney started to develop detectable levels of tubular atrophy and interstitial fibrosis, both unequivocal signs of chronic graft loss [40]. Use of Vectisol^®^ at the time of organ preservation was able to protect the graft from the advance of these lesion processes. As the compound is relatively short-lived (9–10 h), the observed benefits all stemmed from better protection of the cells during IR. While the exact mechanisms of action will require further study, several of the pathways with which resveratrol has been involved have been demonstrated to be linked to the physiopathology of IR [41]. Furthermore, the protection provided by the treatment may also have been due to the cyclodextrin, as the MP study demonstrated that several markers were significantly found to be in the BioCyd group, among which were ASAT and three months of histological lesions. This effect may have been due to the impermeant properties of the compound [42,43], thus lowering the rate of edemas and subsequent cell death, one of the central consequences of IR.

Our results demonstrate the superiority of Vectisol^®^ supplementation over standard preservation solutions. However, one limitation of our study can be found in the static preservation arm, where data analysis is hampered by the low number of animals used, particularly in the Custodiol^®^ and SCOT15^®^ groups. While *n* = 3 is usually considered to be the lowest number of repeats for a test, it is too low to perform proper non-parametric tests, which are required in our setting. However, we feel that a higher number of animals in the UW and Celsior^®^ groups, and confirmation of a similar pattern of effect across solutions, validated this strategy, as we think that more animals would go against the 3R (Replacement, Reduction, Refinement) rule. Another limitation stems from the use of MP, a strategy which is already very effective in protecting the organ against IR [32]; and indeed, the effect of the molecule could not be demonstrated during the early follow-up. Herein, the extended follow-up permitted by our surgical platform was able to discriminate between the groups; however, this questions the relevance of this model in investigating further improvement of the preservation strategy. Indeed, donor demographic evolution predicts a continuous decrease in organ quality, which will require a compounded approach to IR alleviation, combining technology and molecules targeting the multiple aspects of the injury, such as oxidative stress, coagulation, complement, alarmins, and vascular homeostasis. Such investigation will require a more discriminant model, heightening the level of the injury to reflect future organ quality.

## 4. Materials and Methods

### 4.1. In Vitro Experiments

Primary human kidney endothelial cells were used as previously described [44], where ischemia was mimicked by 24 h incubation in a hypoxic atmosphere (95% N_2_, 5% O_2_, Bactal 2 gaz, Air Liquide, Paris, France) at 4 °C in UW or UW + Vectisol^®^ solutions, while reperfusion used regular culture media. Control cells were non-ischemic cells, cultured in regular media in a normoxic atmosphere. Survival was assayed with the Mitochondrial Succinate Deshydrogenase activity (XTT kit, Roche Diagnostic, Meylan, France) following the manufacturer’s guidelines. Reactions were quantified by using a spectrophotometer (Victor3, Perkin-Elmer, Waltham, MA 02451 USA).

### 4.2. Experimental Model

Animal experiments were conducted at the MOPICT (Modélisations Précliniques, Innovations Chirurgicales et Technologiques) platform in Surgères, France, in accordance with the ARRIVE guidelines, from the French Government and the Institutional Comity on the Ethics of Animal Experiments of the Poitou-Charentes (Lusignan, France) (accreditation number of the comity C2EA-84; approval number of the protocol: CE2012-4, 05 July 2012). For this study, we used three-month-old, male, large white pigs weighing 35–40 kg.

For the static preservation model, we used a previously described porcine autotransplantation model in which the kidney was subjected to 24 h cold preservation in the designated solution before re-implantation to the same animal after performing nephrectomy of the contralateral kidney [45]. Four solutions were tested: Celsior^®^, University of Wisconsin^®^ (UW), Custodiol^®^, and Solution pour la Conservation des Organes en Transplantation (SCOT15^®^). Each solution has two groups: Ctr, in which the solution was untouched, and Vect, in which each liter of solution was supplemented with one dose of Vectisol^®^ (1.56 g; containing 2.2 mg trans-resveratrol and 1577.8 mg cyclodextrins). The number of animals per group is detailed in Supplementary Table S1. Animals were followed for one month.

For the machine perfusion model, we designed an auto-transplant porcine model mimicking DCD in which the kidney undergoes one hour of warm ischemia in situ by ligation of renal vessels before collecting and flushing with the Kidney Perfusion Solution^®^ (KPS, Organ Recovery Systems, Diegem, Machelen, Belgium). The organ was then preserved in the Lifeport^®^ machine for 23 h at 4 °C. Finally, the kidney was re-implanted to the same animal after performing nephrectomy of the contralateral kidney [32,33].

Three different groups were performed (*n* = 6 per group):

**BioCtr**: the kidney was preserved in KPS in the Lifeport^®^ machine for 23 h at 4 °C.

**BioCyd**: the kidney was preserved in KPS supplemented with 1557.8 mg/L cyclodextrins.

**BioVec**: the kidney was preserved in KPS supplemented with one dose of Vectisol^®^ (1.56 g; containing 2.2 mg trans-resveratrol and 1577.8 mg cyclodextrins).

Animals were followed for three months.

### 4.3. Biochemistry Analysis

After the transplantation, the animal was put in an individual cage for one week. Urine and blood samples were collected at specific intervals for plasma creatinine, fractional excretion of sodium, serum ASAT, urinary creatinine, and proteinuria analyses using a Cobas bioanalyser (Roche-Diagnostics, Meylan, France). Plasma SOD was measured using ELISA (Bertin Pharma, montigny le bretonneux, France).

### 4.4. Histological and Histochemical Study

Cortical biopsies were collected at Day 7 by using an echo-guided procedure and processes for PAS staining, followed by histological analysis by an anatomopathologist. The degree of histological lesions was determined by semi-quantitatively grading the percentages of lesions by field, where scores were: 0 = no alteration, 1 = lesions < 25%, 2 = lesions between 25–50%, 3 = lesions between 50–100%.

Apoptosis was measured using the terminal deoxynucleotidyl transferase (TdT)-mediated dUTP nick end labeling (TUNEL) assay (Promega, Lyon, France) and quantified as the number of apoptotic cells over the total observed cell ratio. Oxidative stress was quantified using Cell Rox labelling (Thermofisher, Dardilly, France), and expressed as the ratio of a positive signal (green for Cell Rox) over the total signal (blue for DAPI).

The score of histological renal tissue injuries evaluation was obtained in a blinded fashion by an anathomopathologist and a nephrologist on hematoxylin eosin (HES) staining. Histological evaluation of interstitial fibrosis through Sirius red staining, and birefringence visualization using a polarized light to discriminate collagen fiber packing were quantified with ImageJ software.

### 4.5. Statistical Analysis

All the results are shown as mean ± standard deviation (SD). All statistical analyses were performed with R (The R Foundation for Statistical Computing) using Wilcoxon and Dunn’s test, with post-hoc (Kruskal Wallis) multiple comparison post-tests for two-group and three-group analyses, respectively. Outliers were revealed with the Grubb’s test. Results with a *p* value < 0.05 were considered significant.

## 5. Conclusions

In summary, a single dose of Vectisol^®^, a very stable compound with an extended shelf life, was able to significantly improve the performance of the most-used preservation strategies currently used in kidney transplantation. This easy-to-implement strategy can likely be applied to other organs, particularly as we demonstrated compatibility with dynamic preservation, a modality also deployed on the other major organs in transplantations such as for the heart, liver, and lungs. This compound is also a very good candidate for a multi-drug approach to IR in transplantation, an unavoidable development in response to donor demographic evolution.

## Figures and Tables

**Figure 3:**
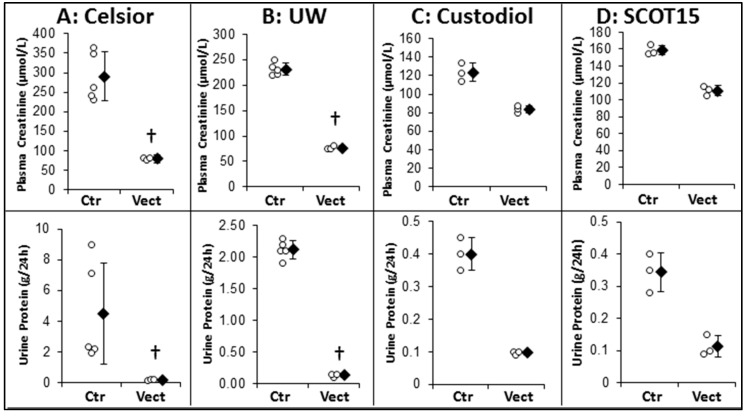
Evaluation of Vectisol^®^ benefits during static preservation: late follow-up. Vectisol was used to supplement preservation solutions in a porcine model of kidney autotransplantation. Solutions used were: (**A**) Celsior^®^; (**B**) University of Wisconsin^®^ (UW); (**C**) Custodiol^®^; and (**D**) Solution pour la Conservation des Organes en Transplantation (SCOT15^®^). Pigs were euthanized after 1 month of follow-up. Shown are: **Top**: plasma creatinine, **Bottom**: urinary protein. Shown are each data point (empty dots) as well as mean ± SD (filled diamonds), statistics (Wilcoxon Test): †: *p* < 0.05 to Ctr.

**Table 1:** Histological evaluation of kidney biopsies at Day 7.

Solution	Celsior		UW		Custodiol		SCOT	
Treatment	Ctr	Vect	Ctr	Vect	Ctr	Vect	Ctr	Vect
Brush Border Loss	2.5 ± 0.1	3.3 ± 0.2 †	3.1 ± 0.1	3.9 ± 0.1 †	2.7 ± 0.3	3.6 ± 0.1	2.2 ± 0.3	3.0 ± 0.1
Endoluminal detachment	2.5 ± 0.1	1.6 ± 0.1 †	1.9 ± 0.2	1.3 ± 0.1 †	1.9 ± 0.2	1.3 ± 0.1	2.2 ± 0.1	1.5 ± 0.1

Cortical biopsies were collected at Day 7 by echo-guided procedures and processes for PAS (Periodic Acid Schiff) staining, followed by histological analysis by an anatomopathologist. The degree of histological lesions was determined by semi-quantitatively grading the percentages of lesions by field: scores: 0 = no alteration, 1 = lesions < 25%, 2 = lesions between 25–50%, 3 = lesions between 51–75%, 4 = lesions between 76–100%. Presented are means ± SD, statistics: †: *p* < 0.05 to Ctr within the same solution.

## Supplementary Materials

Supplementary materials can be found at https://www.mdpi.com/1422-0067/20/9/2268/s1.

## Abbreviations

AMPK	AMP-activated protein kinase
ASAT	ASparagine Amino Transferase
AUC	Area Under the Curve
DCD	Deceased after Circulatory Death
DGF	Delayed Graft Function
HES	Hematoxylin Eosin Staining
HTK	Histidine Tryptophane Ketoglutarate
MP	Machine Perfusion
NO	Nitrous Oxide
PNF	Primary graft Non-Function
ROS	Reactive Oxygen Species
SCOT15	Solution pour la Conservation des Organes en Transplantation
SOD	Superoxide Dismutase
TUNEL	Terminal deoxynucleotidyl transferase (TdT)-mediated dUTP nick end labeling
UW	University of Wisconsin solution
WHO	World Health Organization
