# Supplementary material for: Vectisol Formulation Enhances Solubility of Resveratrol and Brings Its Benefits to Kidney Transplantation in a Preclinical Porcine Model
Source: Int J Mol Sci. 2019 May 8;20(9):2268. doi: 10.3390/ijms20092268 (PMC6540035; doi:10.3390/ijms20092268)
Supplement: Supplementary file 1 [file ijms-20-02268-s001.pdf]

Article

Vectisol Formulation Enhances Solubility of Resveratrol and Brings Its Benefits to Kidney Transplantation in a Preclinical Porcine Model

David Soussi [1,2,3,†], Jérôme Danion [1,2,3,†], Edouard Baulier [1], Frédéric Favreau [4], Ysé Sauvageon [1,2,5], Valentin Bossard [1,2,5], Xavier Matillon [1,6,7], Frédéric Turpin [8], El Mustapha Belgsir [8], Raphaël Thuillier [1,2,3,9,†] and Thierry Hauet [1,2,3,9,10,†,*]

1 UMR Inserm U1082, Inserm Nouvelle Aquitaine, Poitiers F-86021, France; soussi.david@gmail.com (D.S.); jerome.danion@me.com (J.D.); edouard.baulier@etu.univ-poitiers.fr (E.B.); Yse.SAUVAGEON-JAGAILLOUX@chu-poitiers.fr (Y.S.); valentin.bossard988@gmail.com (V.B.); xav.matillon@gmail.com (X.M.); rathuillier@gmail.com (R.T.)

2 Faculté de Médecine et de Pharmacie, Université de Poitiers, Poitiers F-86021, France

3 Service de Chirurgie viscérale et endocrinienne, CHU Poitiers, Poitiers F-86021, France

4 Service de Biochimie et Génétique Moléculaire, CHU Limoges, Limoges F-87042 France; frederic.favreau@unilim.fr

5 Service de Biochimie, CHU Poitiers, Poitiers F-86021, France

6 Faculté de Médecine, Université Claude Bernard Lyon 1, Villeurbanne 69100, France

7 Service d'urologie et de chirurgie de la transplantation, Hospices Civiles de Lyon, Lyon 69003, France

8 BioCydex, 1 rue Georges Bonnet Poitiers 86000, France; fturpin@biocydex.com (F.T.); mbelgsir@biocydex.com (E.M.B.)

9 Fédération Hospitalo-Universitaire SUPORT, CHU de Poitiers F-86021, France

10 IBiSA Plateforme 'plate-forme MOdélisation Préclinique—Innovation Chirurgicale et Technologique (MOPICT), Genesis, INRA, CS 40 052, Surgères 17700, France

† These authors contributed equally to this work.

* Correspondence: thierry.hauet@gmail.com; Tel.: +33-5-49-44-48-29; Fax: +33-5-49-44-38-34

Received: 15 April 2019; Accepted: 05 May 2019; Published: 8 May 2019

Supplementary Materials:

Table S1. number of animals performed in the static preservation study.

Solution.	Celsior		UW		Custodiol		SCOT	
Treatment	Ctr	Vect	Ctr	Vect	Ctr	Vect	Ctr	Vect
n	5	3	5	3	3	3	3	3

Cortical biospsies were collected at day 7 by echo guided procedure and processes for PAS staining followed by histological analysis by an anatomopathologist. The degree of histological lesions was determined using a semi-quantitative grading of the percentages of lesions by field: scores: 0= no alteration, 1= lesions <25%, 2= lesions between 25-50%, 3= lesions between 51-75%, 4= lesions between 76-100%. Presented are means±SD, statistics: *: p<0.05 to Ctr within the same solution.
